# A Peptide-Nanoparticle System with Improved Efficacy against Multidrug Resistant Bacteria

**DOI:** 10.1038/s41598-019-41005-7

**Published:** 2019-03-14

**Authors:** Indrani Pal, Dipita Bhattacharyya, Rajiv Kumar Kar, D. Zarena, Anirban Bhunia, Hanudatta S. Atreya

**Affiliations:** 1 NMR Research Centre, Indian Institute of Science, Bangalore, 560012 India; 2 Solid State and Structural Chemistry Unit, Indian Institute of Science, Bangalore, 560012 India; 3 Department of Biophysics, Bose Institute, Kolkata, 700054 India; 4 Department of Physics, JNTUA College of Engineering, Anantapur, 515002 India

## Abstract

The recent rise of multidrug resistant microbial strains requires development of new and novel therapeutic alternatives. In this study, we present a novel antibacterial system that comprises of modified naturally abundant antimicrobial peptides in conjugation with silver nanoparticles. Further, we propose a simple route to incorporate a cysteine residue either at the N- or C-terminal of the parent peptide. Tagging a cysteine residue at the terminals not only enhances the binding propensity of the resultant peptide with the silver nanoparticle, but also increases its antimicrobial property against several pathogenic bacterial strains including *K. pneumoniae*. The minimum inhibitory concentration (MIC) values of the cysteine tagged nanoconjugates were obtained in the range of 5–15 μM compared to 50 μM for peptides devoid of the cysteines. The origin and mechanism of such improved activity of the conjugates were investigated using NMR spectroscopy and molecular dynamics (MD) simulations. The application of ^13^C-isotope labelled media to track the metabolic lifecycle of *E. coli* cells provided further insights into the system. MD simulations showed that pore formation in membrane bilayer is mediated through a hydrophobic collapse mechanism. The design strategy described herein opens up new-avenues for using biocompatible nanomedicines as a potential alternative to conventional antibiotics.

## Introduction

In recent years, the emergence of multi-drug resistant strains of pathogenic microbes has necessitated the development of new therapeutic alternatives^1^. In this context, antimicrobial peptides (AMPs) have come into focus as a substitute for broad spectrum antibiotics^2,3^. Owing to their ampipathicity, the peptides efficiently target microbial membranes. Given that these peptides are naturally occurring, their biocompatibility is higher when compared to synthetic drug molecules. Despite their rising popularity, the general applicability of these peptides often requires further modifications to enhance their activity when applied as a drug molecule. In this context, tagging of peptides with nanoparticles has been shown to result in improved potency^4–7^. Conjugation of AMPs with nanoparticles has been proposed to result in increasing the local concentration of the peptide at the site of delivery, making the peptide targeting more efficient. In particular, conjugation with silver nanoparticles (AgNP) has the added benefit because of its antimicrobial properties. Thus, combining AgNP with AMP can potentially lead, through a synergistic effect, to a more effective therapeutic solution. The AMP-AgNP conjugates have been shown to exhibit increased stability with activity higher than either the AMP or AgNP considered separately^6^. Previous studies have also helped to signify the role of cysteine residue in forming stable conjugates^5^.

In this study, we adopt such a strategy to modify a potent AMP, Andersonin-Y1 and form its conjugate with AgNPs. The resultant conjugates exhibit nearly 10-fold increase in antibacterial activity against multidrug resistant strains. The microbial strains chosen for the study were chosen from the known infectious ESKAPE pathogens. Amongst the ESKAPE (*Enterococcus faecium*, *Staphylococcus aureus*, *Klebsiella pneumoniae*, *Acinetobacter baumannii*, *Pseudomonas aeruginosa*, and *Enterobacter* species) species, three were chosen~*Klebsiella pneumonia, Pseudomonas aeruginosa* and *Salmonella typhi* (*Enterbacter*). Real time (*in-situ*) monitoring of bacterial cells by in-cell NMR studies together with molecular dynamics studies provide valuable insights into the antimicrobial property. Biophysical characterization of the structural and functional attribute of the resultant conjugates help to elucidate the underlying mechanism involving the targeting of the bacterial membranes.

## Methods

No studies were conducted on humans or human tissues and the study did not involve storage of any human samples. For the hemolysis assay a written informed consent was taken from the volunteer prior to the commencement of the study as per the declaration of Helsinki, 2013 (World Medical Association. World Medical Association Declaration of Helsinki: ethical principles for medical research involving human subjects. JAMA. 2013 Nov 27;310(20):2191–4., 10.1001/jama.2013.281053). The experimental protocols were approved by the Human Ethics Committee of Bose Institute, Kolkata.

### Bacterial Strains

The common laboratory strain of *Escherichia coli* DH5α was used along with three tested members of the ESKAPE (*Enterococcus faecium*, *Staphylococcus aureus*, *Klebsiella pneumoniae*, *Acinetobacter baumannii*, *Pseudomonas aeruginosa*, and *Enterobacter species*) pathogens for the antimicrobial assay. Of these, *Pseudomonas aeruginosa* (ATCC 27853), *Salmonella typhi* (ATCC 14028) and *Klebsiella pneumoniae* (ATCC 13883) were obtained from ATCC (USA).

### Media composition

*E.coli, P. aeruginosa, S.typhi* and *K. pneumoniae* were all grown in Nutrient broth (Himedia) for the antimicrobial assays.

#### Preparation of nano-conjugates

Preparation of nano-conjugate requires several steps which have been discussed in the following sections.

#### Preparation of Silver nanoparticles

Citrate capped silver nanoparticles (AgNPs were synthesized from silver nitrate (AgNO_3_) using sodium borohydride as a primary reducing agent and trisodium citrate acting as both a reducing and capping agent to obtain the diameter of ~10 nm. 2 mM of sodium citrate and 2 mM sodium borohydride were mixed using a magnetic stirrer for 30 minutes at 60 °C. Following this, 1.17 mM silver nitrate was added to the solution and temperature was maintained 90 °C to obtain the nanoparticle solution. Finally the solution was concentrated to 300 μg/ml.

#### Preparation of Peptides

Chemically synthesized Andersonin-Y1 (AY1) [FLPKLFAKITKKNMAHIR], CAY1 [CFLPKLFAKITKKNMAHIR] and AY1C [FLPKLFAKITKKNMAHIRC] were purchased from Hysel India PVT LTD with 95% purity.

#### Preparation of nanoconjugate

The conjugates were prepared by mixing 450 µL of 0.3 mM of peptide (dissolved in H_2_O or buffer) in with 50 µL of 50 µg/mL AgNP at 298 K. Thus, AgNP is diluted to 10 µg/mL.

#### UV-Visible Spectroscopy

The UV-Visible spectra of AgNP-AMP conjugates were recorded on a Shimadzu UV-1800 UV-Vis spectrophotometer with slit width of 1 nm using a quartz cuvette having a path length of 1 cm with a wavelength range of 200–800 nm.

#### Transmission Electron Microscopy (TEM)

The TEM images of silver nanoparticles and AgNP-AY1conjugates were obtained with a Technai T-20 machine operating at a voltage of 200 kV. The samples were prepared using the drop casting method.

#### Calculation for Number of peptides molecules bound with a nanoparticle

The number of peptide molecules binding with one silver nanoparticle (AgNP) was obtained by the following equation as described by Calzolai *et al*.^1^

$${{\rm{N}}}_{{\rm{pep}}}={\rm{0.65}}({{\rm{R}}}_{\mathrm{AgNP}\mbox{--}\mathrm{pep}}^{3}-{{\rm{R}}}_{{\rm{AgNP}}}^{3})/{{\rm{R}}}_{{\rm{pep}}}^{3}$$

where,

R_AgNp–pep_ = Radius of conjugated nanoparticle (7 nm).

R_AgNp_ = Radis of AgNP (6 nm).

R_pep_ = diameter of peptide (0.6 nm).

#### Scanning electron microscopy (SEM)

Bacterial cells (Gram-negative BL21 (DE3) *E. Coli* cells) were inoculated in different tubes with 500 μl of Luria-Broth (LB) medium from single colonies for each sample and were allowed to grow until it reached an optical density of 0.3 as measured at 600 nm and treated separately with different samples. After addition of AY1, AgNP or AgNP-AY1 conjugates, the cells were grown further for 1 hr and pelleted at 10,000 rpm for 2 min. The pellets were washed with phosphate buffer saline three times and pre-fixed with 2.5% glutaraldehyde. The pre-fixed cells were were taken on a silicon wafer and kept for vacuum drying and subjected to gold-coating using an ion sputter. The images of the cells were obtained by a FEI Sirion XL30FEG SEM under high voltage varying between 200 kV and 300 kV.

#### Preparation of Membrane Mimics

*For NMR Spectroscopic and CD Spectroscopic studies*: The well-studied bacterial membrane mimic model were prepared through large unilamellar vesicles (LUVs) which were obtained from POPC (1-Palmitoyl-2-oleoyl-sn-glycero-3-phosphocholine) and POPG (1-palmitoyl-2-oleoyl-sn-glycero-3-phosphoglycerol) taken in the molar ratio of 3:1. First the thin flim was prepared by drying the lipid mixtures which were in chloroform into a stream of nitrogen gas, followed by lyophilisation for overnight. Then 300 µl of a 100 mM phosphate buffer (pH 6) was added to the lipid film to make a final concentration of 5 mg/mL. Following this, it was freeze-thawed followed by rigorous vortexing and then the lipid mixture was extruded 25 times through a 100 nm polycarbonate nucleopore filter membrane so as to obtain LUVs of approximately 100 nm diameters.

*For Fluorescence studies*: For fluorescence studies, the bacterial membrane mimicking LUVs were prepared using POPE (1-Palmitoyl-2-oleoyl-sn-glycero-3-phosphoethanolamine) and POPG (1-palmitoyl-2-oleoyl-sn-glycero-3-phosphoglycerol) in a molar ratio of 7:3. A thin film was prepared using a stream of Nitrogen. The film was hydrated using 300 µl of a 10 mM phosphate buffer containing 70 mM 6-carboxyfluorescein dye, resulting in a final concentration of about 2 mg/mL. After thorough vortexing, extrusion through a 100 nm polycarbonate membrane resulted in the formation of LUVs with diameters of 100 nm. The resulting suspension was passed through a centrisep column to remove the excess dye from the buffer solution. This ensured that most of the dye molecules remained entrapped within the LUV interior.

#### Fluorescence Spectroscopy

The fluorescence emission data at 520 nm was obtained using a Hitachi F-7000 FL spectrometer with excitation wavelength of 495 nm. The excitation and emission slit widths were adjusted to 5 nm and a PMT detector voltage of 700 V was used for collecting the data. The peptides alone or AgNP-AMP were added in increasing concentrations (5 µM to 35 µM) to the sample containing large unilamellar vesicles (LUVs). The LUVs were incubated for about 7 minutes for each concentration of peptide added to the solution, before acquiring the emission spectrum to monitor the increase in fluorescence caused due to the leakage of the dye from the vesicle interior (denoted as ‘I’). The maximum fluorescence intensity (I_100_) of the dye used in the assay was determined by the addition of 5 μL of 10% Triton X-100, which can be considered to have caused 100% leakage of the dye due to disruption of all the vesicles in the sample volume. Therefore, percentage of leakage caused by the peptides can be calculated using the following equation:

$$ \% {\rm{leakage}}=[({\rm{I}}-{{\rm{I}}}_{{\rm{0}}})/{({\rm{I}}}_{100}-{{\rm{I}}}_{{\rm{0}}})]\times 100$$

where, I_0_ is the fluorescence intensity of LUVs alone; I and I_100_ are the fluorescence intensity of the dye in the presence of peptide and Triton X-100, respectively.

All data were collected in triplicates and plotted with the standard deviation as the error bars.

#### Minimum Inhibitory Concentration determination

The microtiter broth dilution method was used to determine the MIC as previously described^8^. Overnight cultures of the mentioned bacterial strains were washed twice and re-suspended in either 10 mM phosphate buffer, pH 7.4, and further diluted to 10^5^ cfu/ml. The peptides and the conjugates were prepared to a stock concentration of 1 mM. 50 µl of microbial suspension was added to each corresponding well of a 96-well microtiter plate (polypropylene) together with 50 µl of peptide solution. The wells each had peptide concentrations adjusted to a range- increasing from 1 µM to a maximum of 100 µM. The plate was subsequently incubated at 37 °C with constant shaking for 3 h. Following this incubation, 200 µl of media was added and allowed for further incubation for 24 to 48 h. The MIC was obtained by recording the O.D of each of the wells at 630 nm. The values were normalized with respect to the negative control. 10 mM Polymyxin B was used as a positive control that showed about 85% of killing in each strain of bacteria tested. Microbial suspension in the absence of peptide was used as the negative control. All data were collected in triplicates and plotted with the standard deviation as the error bars.

#### Haemolysis assay

Freshly collected human blood cells were pelleted down by centrifugation at 4000 g for 10 minutes at 4 °C and washed thrice with PBS (pH 7.4). The pellet was resuspended to obtain a final 1 × 10^8^ erythrocytes/mL suspension. This suspension was treated with equal volumes of increasing concentrations of the peptides up to 100 µM at 37 °C in incubator shaker for 1 hour. The treated samples were again centrifuged at 900 × g for 10 min at 4 °C and the absorbance of supernatant was measured at 540 nm to quantify RBC lysis. 2% Triton X 100 was used as a control which caused complete (100%) lysis. Finally percentage hemolysis was calculated using the following equation:

$$ \% \mathrm{of}\,{\rm{haemolysis}}=({{\rm{O}}}_{{\rm{p}}}-{{\rm{O}}}_{{\rm{b}}})/({{\rm{O}}}_{{\rm{m}}}-{{\rm{O}}}_{{\rm{b}}})\times 100$$

where, O_p_ = optical density of the sample upon treatment with a given peptide concentration,

O_b_ = optical density of buffer and

O_m_ = optical density of Triton × 100.

All the data were collected in triplicates and were plotted along with the standard deviations.

#### Circular Dichroism (CD) Spectroscopy

The CD spectra were recorded on a JASCO CD Spectrometer to study the secondary structure of the AY1 in free and membrane bound form and scanned from 190 to 250 nm. The samples were prepared in phosphate buffer (pH = 6). 0.3 mM peptide was titrated with increasing concentrations of PG/PC LUV and the spectra were recorded at room temperature (25 °C) with accumulations of three scans with a data interval of 1 nm. The ellipticity in milli degrees was plotted against wavelength in nm.

#### NMR Spectroscopy

All NMR data were recorded at 298 K and 288 K on a Bruker Avance III 800 MHz NMR spectrometer equipped with a cryogenically cooled triple resonance probe. The sequence specific resonance assignment of the peptides/conjugates was carried out by analysing in concert 2D ^1^H-^1^H Total correlation spectroscopy (TOCSY) and 2D ^1^H-^1^H nuclear Overhauser spectroscopy (2D NOESY). A mixing time of 80 ms was used in the TOCSY experiment with 16 transients and 256 complex points in the indirect dimension. The 2D NOESY spectra were recorded using a mixing time of 150 ms, 32 transients and 512 complex points in the indirect dimension with an overall measurement time of 9 hours. The 2D [^13^C, ^1^H] HSQC was acquired with 16 transients and 256 complex points, resulting in a measurement time of 3 hours.

For in-cell NMR studies the 1D version of a 2D ^13^C-^1^H HSQC (i.e., the first increment) was recorded with 512 scans. The *E.coli* cells were grown in minimal media containing ^13^C labeled glucose until the cells reaches OD = 0.6. Then the cells were pelleted and resuspended in phosphate buffer. These cells were added to 0.5 mM each peptide and nano-conjugate solution separately and the cell metabolites were monitored through NMR at different time points: 0^th^, 0.25^th^, 0.5^th^, 1^st^, 2^nd^, 3^rd^, 4^th^, 5^th^, 6^th^ and 12^th^ hour. The intensity of the peaks in 3–4 ppm (corresponding to glucose) were integrated in each of the 1D (first increment) of the ^13^C-^1^H HSQC spectrum after Fourier transformation and the intensity vs time were plotted.

#### Peptides Structure calculation

After resonance assignment using 2D TOCSY and 2D NOESY the peptide structure was calculated. The upper bound distance constraints were calculated with respect to the peak integrals. A 2.0 Å distance was fixed as the lower distance limit. The calculated distances were classified as strong (2.5 Å), medium (2.6–3.5 Å) and weak (3.6–5.0 Å) for structure calculation using CYANA. It is worth mentioning that the folding of the structure depends mainly only on medium-and long-range NOEs. The backbone dihedral angles Ф and ψ were varied from −30 to −120 for all non-glycine residues to reduce conformational search. The refinements were performed in some few consecutive steps. Among the 100 calculated structures 20 lowest energy structures were chosen to depict the ensemble structure. For visualizing and quality checking of the 3D structural models Procheck, PyMOL, and MOLMOL were used. The structures of the three peptides (AY1, AY1C and CAY1) in membrane were deposited in Protein data bank (PDB) having the following codes, respectively: AY1:PDB ID 5YKK, CAY1:5YKQ, AY1C:5YKL.

#### Molecular dynamic Simulation

*MD Simulation system*: The three-dimensional coordinates of peptide AY1, determined in solution using NMR technique was chosen for MD simulations, of which the lowest energy structure was used for coarse grain molecular dynamics (CG-MD) simulation. Based on the experimental evidence, it was hypothesised that silver nanoparticle conjugated peptides dissociate after entering the membrane. Furthermore, the peptides undergo self-aggregation within the lipid environment, thereby forming a channel or pore across the membrane architecture. The objective of using CG-MD in the present work was thus to trace the biomolecular dynamics and collect evidence to justify that peptide AY1 tends to undergo association and creates a channelized pore.

The simulation system was thus prepared using 20 units of peptide AY1 (all-atoms) arranged at a minimal distance of 10 Å separation from each other, using maestro module of Schrodinger: Schrödinger, L. L. C. “Schrödinger Release 2014–1”. *Maestro, New York, NY* (2014). The modelled system was saved in PDB file format, which was then processed using CHARMM-GUI membrane builder^9^. The construct also included a bilayer of heterogeneous lipids, POPG: POPC in 1:3 ratio, in a rectangular box type. The length of Z-axis was based on water thickness, in which minimum water height on both side of leaflet was set to 17.5 Å. The insertion method was used to place the peptide system into the bilayer (~10.0 Å from the edge surface). The total number of lipids in the upper and lower leaflet comprises of 648 lipid moieties and the lipid concentration on both leaflets were symmetrical. The peptide system with +67 charge was neutralised with Cl- counter ions and was solvated. Representative overview of the CG models for POPC, POPG, and peptide AY1 is show in the supporting information (Fig. S6).

*MD parameters*: The CG-MD simulation were performed using GROMACS package v5.0.6, with the help of martini CG forcefields^10^. Briefly, forcefields used within the system include martini v2.2 for protein and v2.0 for lipids and ions. The Lennard Jones potential and coulomb potentials were shifted to zero using range of 0.9–1.2 nm and 0–1.2 nm, respectively. Importantly, the peptides, lipids, water molecules, and ions were coupled independently, and the system temperature was set to be 310 K with velocity rescaling thermostat and 1 bar semi-isotropic pressure coupling using Parrinello-Rahman barostat. The periodic boundary condition and non-bonded interaction was applied to the system. An integration time step of 20 fs for the production MD run with non-bonded interaction cutoff 1.2 nm was used for the CG-MD simulation. The system was equilibration for a time scale of 50 ns, and that production run was continued for 1 µs time scale. The MD trajectory was analysed using VMD program, v1.9.2, interpreted using gromacs modules, and visualised using PyMOL (http://www.pymol.org/).

## Results

**Figure 1:**
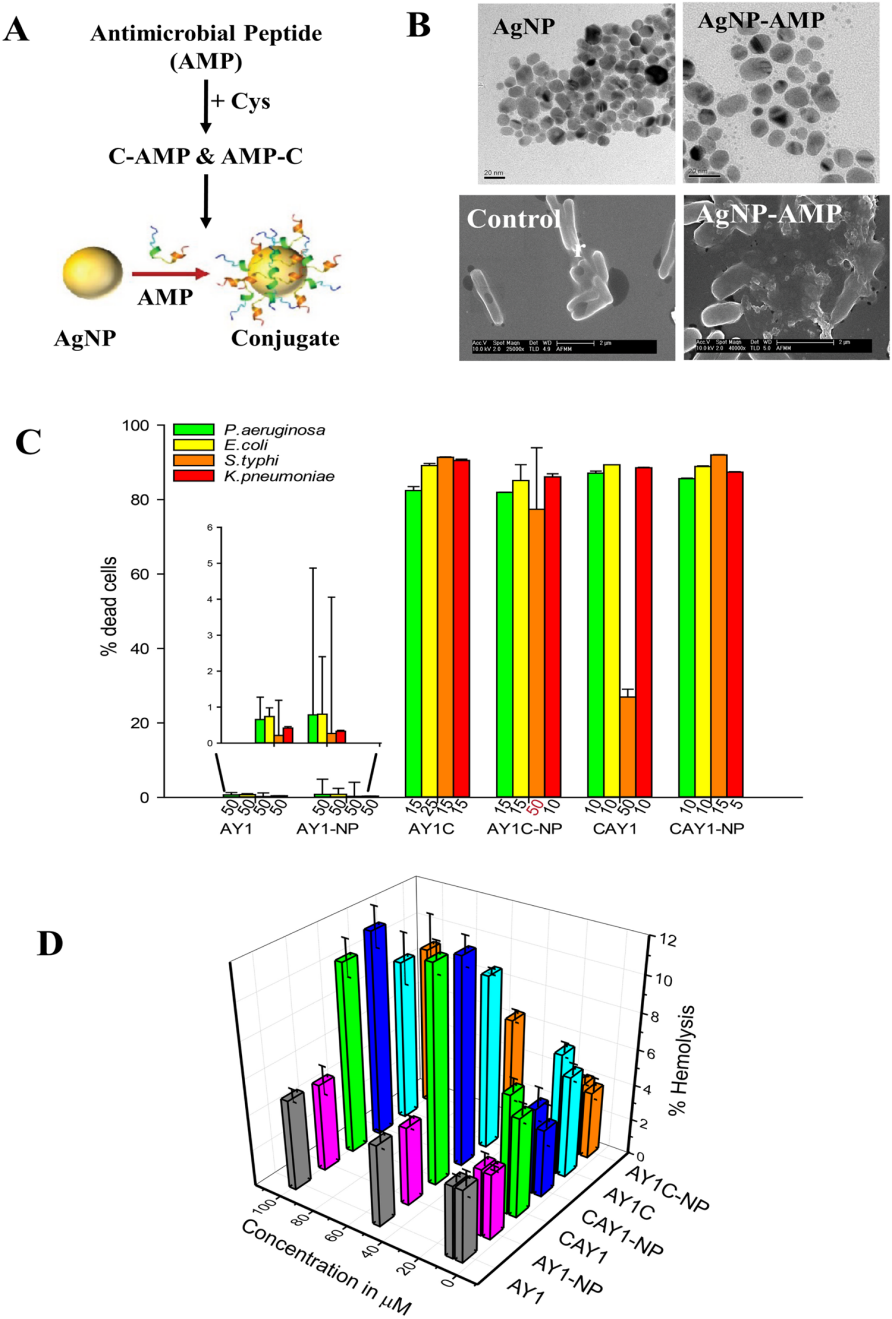
(**A**) Design strategy of peptides; (**B**) (i) TEM images of AY1 conjugated silver nanoparticle (AgNP-AMP) (ii) SEM images of untreated and AY1-AgNP treated *E.coli* cells; (**C**) Peptide concentrations showing at least 80% killing (except CAY1, showing less than 40%), error bar represents the standard deviations of the individual data for three repeats. As a control, we evaluated the MIC for AgNP alone over a concentration range of 10–15 μg/ml (used in the formation of nano-conjugate) and no cell inhibition was observed (n = 3). (**D**) Percentage of haemolysis with increasing concentrations of peptide and their conjugates (n = 3).

Anderosin-AY1^11^ (FLPKLFAKITKKNMAHIR- hereafter referred to as AY1) with five positively charged residues is devoid of any cysteine in its sequence. It has been observed that conjugation of AgNPs with a peptide containing cysteine confers stability to the nano-peptide conjugate^6^. We, therefore, constructed two new peptides by adding cysteine to the N-terminus as CFLPKLFAKITKKNMAHIR (CAY1 peptide) and the C-terminus resulting, respectively, in FLPKLFAKITKKNMAHIRC (AY1C peptide) [Fig. 1A]. Silver nanoparticles of 10 nm size were prepared by citrate capping (see Experimental Sections 1–4 of Supporting Information) and used to form the corresponding conjugates for the three peptides, namely, AY1-AgNP, AY1C-AgNP and CAY1-AgNP. The nanoconjugates were first monitored using UV-visible spectroscopy, which showed an absorbance at 400 nm corresponding to an average particle size of 12–14 nm [Fig. S1, Supporting Information]. This was further verified using transmission electron microscopy (TEM) [Fig. 1B, upper pane].

To obtain further insights into the dynamics of interaction between the nanoparticle and the peptides, we performed NMR titrations for each of the peptides with silver nanoparticles [Fig. S2]. In case of AY1, positively charged residues such as K4, K8, K11, K12, H16 and R18 exhibit significant changes in chemical shifts in the fast exchange phenomena upon conjugation with the negatively charged nanoparticles. Two dimensional (2D) [^13^C-^1^H] HSQC spectra acquired for AY1 and the conjugate also clearly showed the chemical shift changes for side-chain ^1^H and ^13^C atoms for the K11, K12, H16 and R18 residues upon binding to the nanoparticle surface (Fig. S2). Similar chemical shift effects exhibiting fast exchange on the NMR time scale was obtained for AY1C and CAY1 when bound to the nanoparticle and the free peptide [Fig. S2]^6,12–14^.

The peptides and their nanoconjugates exhibited membrane directed activity against bacterial cells. The bactericidal activity of the peptides and its nanoparticle conjugates were examined by measuring the minimum inhibitory concentration (MIC)^14^, against pathogenic bacterial strains. The functional interaction was further studied using real-time in-cell NMR spectroscopy. Scanning Electron Microscopy (SEM) revealed the altered bacterial cell surface morphology followed by membrane rupture upon treatment with the nanoconjugates (Fig. 1B, lower panel). Fluorescence based dye-leakage assays was used to measure the membrane directed activity of the peptides/nanoconjugates using bacterial membrane mimics^15^.

The estimation of MIC values correspond the improved potency of the nano-conjugates when compared to the parent peptides. The conjugates: AY1C-AgNP and CAY1-AgNP exhibit improved potency against all the tested strains (Fig. 1C). Figure 1C represents the concentrations of C-tagged peptide and the nano-conjugates corresponding to which at least 80% of bacterial killing was obtained. The MIC values were found to be 15 μM and 10 μM, respectively, against *P. aeruginosa* while they showed 12 μM and 10 μM, respectively against *K. pneumoniae*. Similarly, CAY1 and CAY1-AgNP showed similar MIC_80%_ of 10 μM against *P. aeruginosa* and *E.coli* cells. Although CAY1 did not show potential activity in case of *S. typhi*, conjugation with AgNP increased the antimicrobial activity (MIC_80%_) of 15 μM. The CAY1-AgNP conjugate was also very effective against multi-drug resistant *K. pneumoniae* with a MIC_80%_ of 5 μM. As a control, we evaluated the MIC for AgNP alone over a concentration range of 10–15 μg/ml (used in the formation of nano-conjugate) and no cell inhibition was observed. N-terminal tagged AY1 showed less than 40% killing against *S.typhi* cells even upto 50 μM which increased to more than 80% in case of NP tagging of the same when tested for only 10 μM. Nevertheless the modified peptide showed increased effective killing of all the tested strains when compared to 50 μM of the parent peptides. Table 1 represents the average MIC_80%_ values for the parent peptide and its nanoparticle conjugates tested against four different bacterial strains.

**Table 1:** Average MIC values (in μM) of the peptides and their nanoconjugates against four different bacterial strain (see Fig. 1C).

	AY1	AY1-NP	AY1C	AY1C-NP	CAY1	CAY1-NP
*P. aeruginosa*	~50	~50	15	15	10	10
*E. coli*	~50	~50	17	12	10	10
*S. typhi*	NA	NA	15	15	NA	15
*K. pneumoniae*	NA	NA	12	10	7	5

To evaluate the modified peptides as therapeutic agents, hemolytic assays on human blood samples were performed. This assay enabled us to quantify the hemoglobin release in the plasma as a consequence of RBC membrane lysis of the membrane directed activities of the tested peptides. Interestingly, the peptides and their nano-conjugates showed negligible hemolytic activity against RBCs^16–18^ with a maximum value of less than 12%, tested up to a concentration of 100 μM (Fig. 1D). AY1 and its nano-conjugate, AY1-AgNP was non-toxic, with only 5% of cell lysis tested upto a concentration of 100 μM. Almost similar result was obtained for AY1C-AgNP and CAY1-AgNP manifesting in 8.9% and 11.4% haemolytic activities, respectively. Thus these results cumulatively suggest the peptide efficacy with low cytoxicity against the host cells.

**Figure 2:**
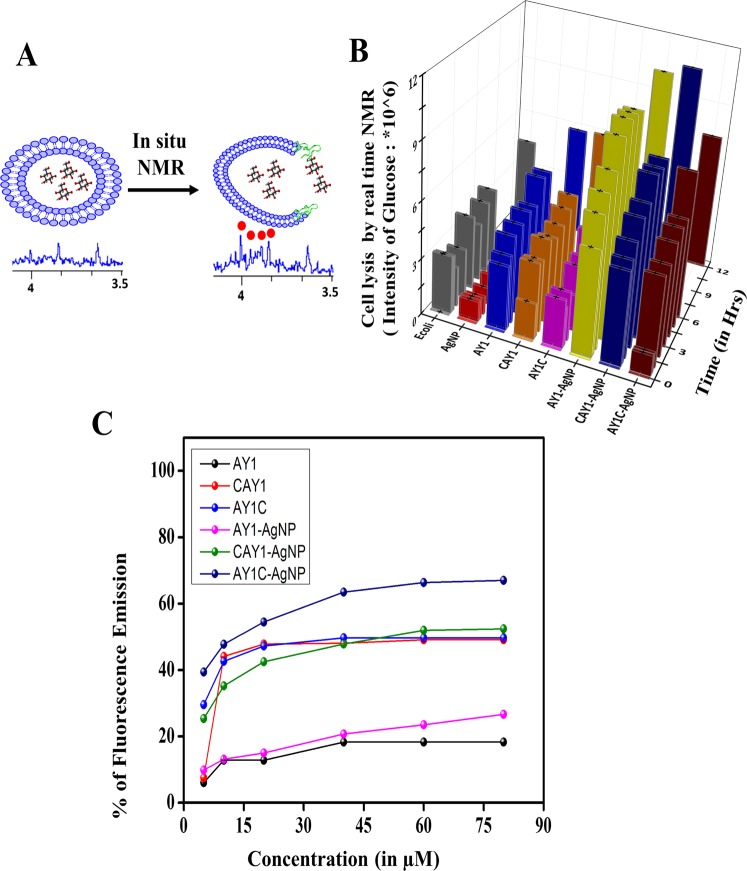
(**A**) Schematic depiction of “*in cell*”-NMR experiment carried out to probe the bacterial cell disruption; the red dots indicate the NMR signals of glucose. (**B**) Plot of glucose intensity v/s time in real time as observed from in-cell NMR spectra; the noise level in the NMR spectrum were taken as the error in the estimation of the signal intensity and (**C**) Fluorescence based dye-leakage assay showing the percentage of membrane leakage initiated by the peptides and the nano-conjugates against the bacterial membrane mimicking Large Unilamellar Vesicles (7:3 POPE/POPG). Increase of fluorescence emission due to dye leakage as a function of peptide concentration. AY1C-AgNP showed the maximum leakage of ~70% at a concentration of 60 μM. All data were collected in triplicates and plotted with the standard deviation as the error bars.

In order to monitor and quantify the rate of bacterial cell lysis by the AMP-AgNP conjugates, we performed a real-time (*in-situ*) in-cell NMR experiment by acquiring the one-dimensional (1D) ^1^H spectra of *E. coli* cells at regular intervals of time (Fig. 2A). The cells were grown in a ^13^C labelled medium. The NMR signals of small molecule metabolites such as glucose emerging from ruptured cells were monitored using the 1D version of a 2D ^13^C-^1^H heteronuclear single quantum coherence (HSQC) experiment. The assumption in this study was that the metabolites inside the cell have restricted freedom of movement resulting in broadening of their NMR signals. However, upon release from the cell, their resonance lines in the spectrum become sharp and grow in intensity as more cells get ruptured. The intensity plot for glucose as a function of time is shown in Fig. 2B for both untreated *E. coli* cells and cells treated with 0.5 mM of AY1, AY1C, CAY1, AY1-AAgNP, AY1C-AgNP, CAY1-AgNP and 5 μg/ml AgNP. The *in-situ* NMR data indicate a higher rate of cell rupturing initiated by the nanoconjugates, AY1-AgNP, AY1C-AgNP and CAY1-AgNP when compared to their parent peptides. In contrast to the untreated cells, after 6 hours of treatment with AY1C-AgNP or CAY1-AgNP, the glucose intensity increases up to ~9–11 × 10^6^. But for the same, after treatment with the peptide or AgNP alone reaches only up to an intensity <3 × 10^6^.

Thus, the activity of the nanoparticle-conjugated peptide is more than the sum of the activities of the peptide and the nanoparticle taken separately. This can be attributed to the AgNP mediated delivery of a large number of peptide molecules close to the bacterial cell membrane along with the enhanced stability of the NP in the presence of the peptides as discussed below. Fig. S3 (Supporting Information) shows an overlay of the spectra acquired at the 1^st^ and the 6^th^ hour depicting the stability of peptide nanoconjugates over time, in the cellular environment with increasing metabolites. Our study, thus, for the first time gives an insight into the real-time cell monitoring of action for antibacterial agents. The method employed using isotope labelled glucose, further opens up a new window to interpret the metabolites concentration over time upon release from the ruptured cells.

The membrane disruptive activity of the peptide was further exemplified using the fluorescence-based dye-leakage assay. The assay involved measuring the increase in fluorescence intensity for the dye, 6-carboxyfluorescence, upon leakage from the membrane mimicking large unilamellar vesicles (LUV). The leakage of the membrane is the directed activities of the peptides or the nanoconjugates^19^. The dye initially has low fluorescence intensity when entrapped within the vesicle interior. Upon rupturing the membrane by the peptides or their conjugates, the dye gets released from the LUVs, resulting the increment of fluorescence intensity (Fig. 2C). The study reveals the least dye leakage of ~18% were obtained for parent peptide at a concentration of 80 μM. However, the fluorescence intensity increases to about 26% for its conjugate, AY1-AgNP. But the designed peptides AY1C and CAY1 showed the percentage of leakage of ~50% at the same concentration (80 μM) while it increased further to 67% and 54%, for AY1C-AgNP and CAY1-AgNP, respectively [Fig. 2C, Table S2].

Insights into the origin of enhanced activity of the conjugates were obtained by analysing the NMR derived 3D structures of the peptides in the absence and presence of model membrane mimics. The sequence specific resonance assignment^20^ of all peptides was carried out in absence and presence of 3:1 POPC/POPG LUVs and the 3D structures were calculated using the program CYANA^21^, based on the NOEs observed in 2D nuclear Overhauser effect (NOE) spectroscopy (complete details of the resulting statistics/quality are provided in Table S1 of Supporting Information).

**Figure 3:**
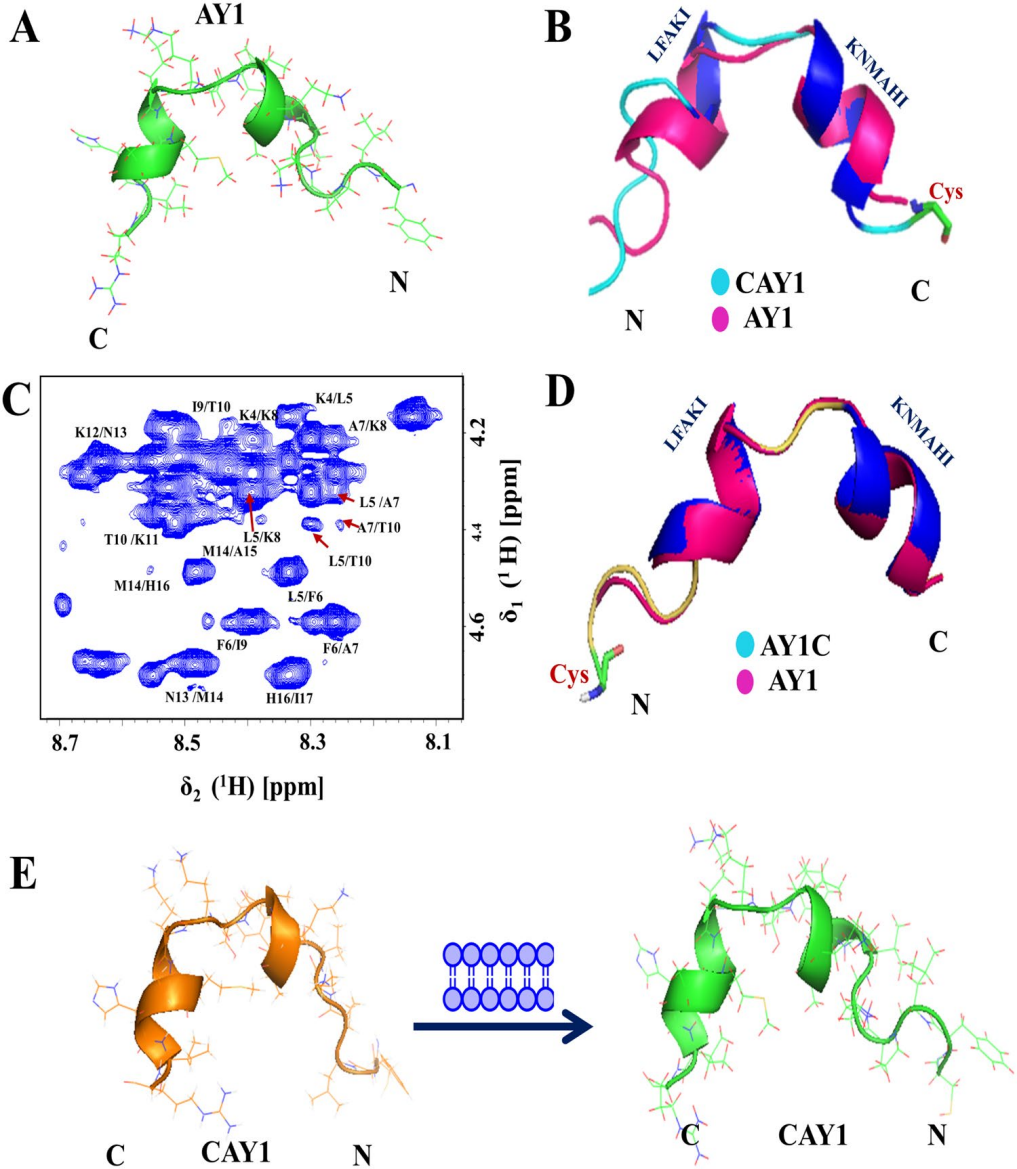
NMR derived structures of AgNP-AMP conjugates of (**A**). Anderosin-Y1 (AY1) [PDB ID 5YKK]. (**B**) Overlay of Anderosin-Y1 (AY1) [in pink] with designed peptide CAY1[PDB ID 5YKQ] [in cyan]. (**C**) The fingerprint region NOEs of AY1-AgNP. (**D**) Overlay of Anderosin-Y1 (AY1) [in pink] with designed peptide AY1C [PDB ID 5YKL] [in yellow] in presence of 3:1 POPC/POPG LUVs and (**E**) schematic depicting active form of CAY1 with and without membrane.

First, the structure calculation of free AY1 in solution was attempted. It was found that AY1 is unstructured in solution, devoid of any regular secondary structural elements. Next, ^1^H NMR titration of AY1 with the LUV was carried out (Fig. S2), which revealed broadening of a few peaks indicating binding of the peptide/conjugates to the membrane. Circular Dichroism experiments were also carried out with 0.5 mM AY1 and AgNP-AY1 being titrated with 5 mg/ml LUV. Upon LUV binding, a helical structural uptake is initiated for the AY1 peptide (Fig. S8). This was further supported by 2D NOESY experiments carried out to derive the structure of the free and membrane bound peptides. A significant number of NOEs were observed for AY1 in presence of LUVs [Fig. 3C].

The membrane bound structure of AY1 reveals a “helical hairpin” (helix-loop-helix) structure^22,23^. There are two helical turns observed in the F6-I9 region and in the K12-H16 region [Fig. 3A]. Figure 3C represents the assigned NOEs of the alpha helical region of the membrane bound AY1-AgNP. These NOEs are clear indication of the alpha helical turns present in the peptide. The structure of AY1-AgNP was found to adopt a similar structure in the presence of the membrane [Fig. 3A].

Figure 3B,D, respectively, depict the overlay of 3D structures of CAY1-AgNP and AY1C-AgNP in the membrane with AY1. In the membrane bound form, the helical turns (highlighted in blue) for AY1C was (Fig. 3B,D) observed from L5 to I9 and K12 to I17 (Fig. S4 TOCSY assignments) whereas for CAY1, they were observed from L6 to I10 and K13 to I18. These two regions were connected by a loop. In the helical hairpin structure of all membrane bound peptides/conjugates, interestingly, the side chains of all positively charged residues such as lysine, histidine, and arginine are exposed on one side of molecular surface. Similarly, the side chains of the hydrophobic residues including phenylalanine, leucine, isoleucine, methionine, cysteine segregate on the other surface, thereby giving rise to an amphipathic structure. Fig. S5 represents the ensemble of 20 structures for each of the AgNP-bound peptides. It is worth mentioning that the addition of cysteine moieties at either of the two terminals of parent-peptides does not affect the corresponding secondary structures much.

Notably, in contrast to AY1, the modified peptide CAY1 adopted some folded conformation even in aqueous solution (Fig. 3E). Both the structures (structure in aqueous solution and in membrane) were very similar. From a structural perspective, the peptide already possesses the membrane-active state in the free form. The presence of a structure in the free form, which is similar to the membrane bound form, would involve lesser energy costs during interaction. This perhaps explains the enhanced action of the designed peptides and their corresponding conjugates (as shown in Figs 1 and 2).

**Figure 4:**
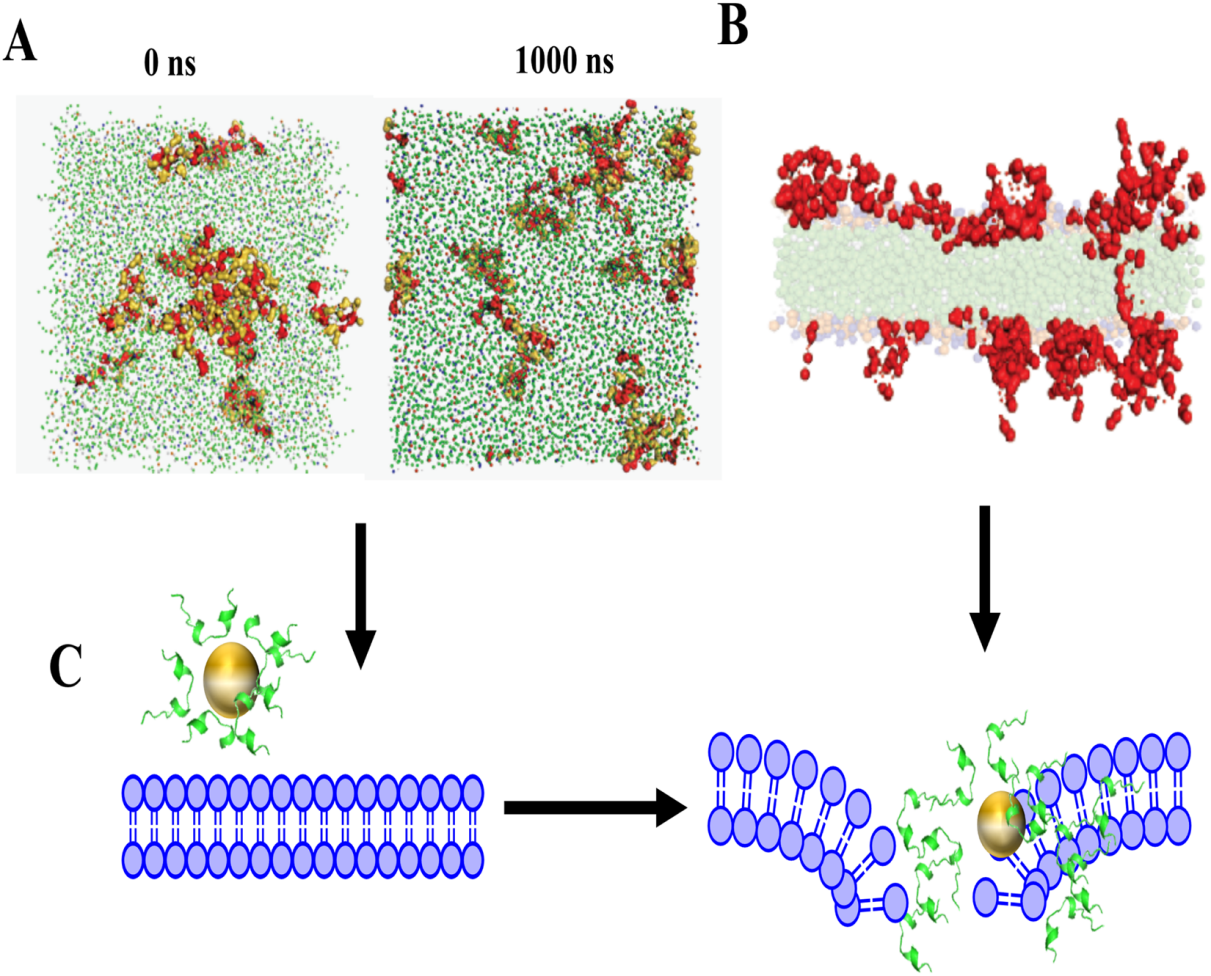
(**A**) Snapshot of CG-MD system at 0 ns and 1000 ns. (**B**) MD model depicting the pore formation by the AY1 peptide assembly and (**C**) Mechanistic hypothesis of cell membrane rupture by nano-conjugates.

We took the help of molecular dynamics simulation to understand the membrane pore forming phenomena^9,10,24,25^. Experimentally, the number of peptides bound to one AgNP particle was estimated to be ~200 (see Methods Section)^6,11,12^. To understand the mechanism of action of the nano-conjugate we performed the coarse grain simulation study with conjugated AY1. The initial system built for our coarse grained MD (CG-MD) simulation comprised randomly placed 20 units of peptide AY1 in lipid bilayer of 3:1 POPC/POPG [see SI, Fig. S6]. The hypothesis of our work was based on the fact that the peptides disrupt the membrane architecture, after getting detached from AgNP surface, possibly mediated via electrostatic and/or hydrophobic interaction. In order to render a better representation within the course of MD trajectory, the hydrophobic residues Phe1, Leu 2, Leu 5, Phe 6, Ile 9, His 16, and Ile 17 were marked to trace the hydrophobic collapse (Fig. 4A,B). The snapshots corresponding to 0 ns and 1000 ns reveal the change in orientation which rules-out the hydrophobic, self-association related mechanism [Fig. 4A]. Figure 4B represents the pore formation through the hydrophobic patches of the peptide inside the membrane. The simulation events are shown in a movie (Supplementary material and Fig. S7) and a mechanistic hypothesis corresponding to the analysis of CG-MD simulation is shown in Fig. 4C.

## Discussion

In recent years, different criteria have been proposed and adopted for the effective design of antimicrobial peptides with increased structural stability and enhanced microbicidal activities^26,27^. Nano-particle tagging is one such effective strategy to modify parent antimicrobial peptides to increase their therapeutic properties. Liu *et al*.^4^ studied the potency of the nano-conjugates from different perspectives such as size and shape and Alarcon *et al*.^5^ have shown the importance of cysteine in the conjugation process. In our previous study, we found that the cysteines are directly involved in the interaction of the peptide with AgNP^6^. The peptide is weakly bound to the AgNPs resulting in a dynamic exchange from the surface of the AgNPs without undergoing any conformational change. This weak interaction is useful particularly in the case of anti-microbial peptides where the charge and structure of the peptide is crucial for its activity^28,29^. A strong interaction with AgNPs can perturb the structure of the AMPs and reduce their activity. Thus, the balance of strong interaction/stability is a key feature that was considered for the design of new conjugates. The stability of the AgNP-AMP conjugate arises from steric repulsion^6,11^. The stability is enhanced if AgNPs are conjugated with polypeptides, wherein the steric repulsion between proteins at close distance prevents nanoparticles from aggregation^6,11^. The long term stability of the conjugate helps in recycling the AgNP-AMP conjugate multiple times as shown recently for AgNP-Ubiquitin conjugates^6,11^. In case of the AgNP-peptide conjugate presented here, nearly 200 molecules of peptides bind to the AgNP surface through electrostatic interactions, which results in a higher concentration of positive charges. The peptide has relatively weaker interaction with the silver nanoparticles when compared to the membrane affinity. Thus delivery of the nano-conjugates enable increased local concentration of the peptide for its membrane directed activity. Furthermore, the stability of AgNP affects the overall efficacy of the peptide conjugates being more than just the sum of the activities of the peptide and the nanoparticle, separately.

During the early equilibration event (as revealed by MD simulations), the random spatial arrangement of peptides is reflective, whereas, with time, peptide gets distributed and aligned to both leaflets. Such alignment also suggests that there exists interaction of peptide with negatively charged phosphate head groups of lipid moieties as well as with water molecules. In the nanoconjugate forms, the core of peptide bilayer containing the acyl chains of lipids is expected to support the initial hydrophobic interactions. However, this driving force cannot be considered conclusive, as majority of peptides have interaction with outer membranous portion. The peptide detaches from the AgNP surface due to the relatively stronger electrostatic interaction between the negatively charged phospholipid head groups of the membrane, forming a pore in the membrane by hydrophobic interaction. Figure 4C schematically depicts a possible mechanism of action of the AgNP conjugated peptide. Subsequently, the hydrophobic residues form a pore through interaction with the hydrophobic tails of the membrane. Once the pore is formed, the silver nanoparticle can enhance bacterial cell death by attaching to its DNA^30^. The modified peptides exhibit higher activity compared to the unmodified parent peptide. This is due to the fact that the modified peptides and the corresponding nano-conjugates adopt similar structures both in the free or in the membrane bound form, making it an energetically favourable process for the peptides/conjugates to bind to the membrane.

In this study, the mechanistic interpretation of nanoparticle-peptide conjugate system in light of its three-dimensional structure and real-time monitoring of cells by NMR spectroscopy helped the in-depth analysis of the action of the conjugates. The lowered minimal inhibitory concentration (80%) for the conjugates against some super bugs such as *K. pneumoniae* provides a promising lead in the area of nanomedicine. This brings out the applicability of AMP nanoconjugates as a new horizon for biocompatible antimicrobial agents with enhanced activity.

## Supplementary information

Molecular Dynamics Video

A Peptide-Nanoparticle System with Improved Efficacy against Multidrug Resistant Bacteria
