# Supplementary material for: A Peptide-Nanoparticle System with Improved Efficacy against Multidrug Resistant Bacteria
Source: Sci Rep. 2019 Mar 14;9:4485. doi: 10.1038/s41598-019-41005-7 (PMC6418133; doi:10.1038/s41598-019-41005-7)
Supplement: Supplementary file 2 — A Peptide-Nanoparticle System with Improved Efficacy against Multidrug Resistant Bacteria [file 41598_2019_41005_MOESM2_ESM.docx]

Supporting Information

**A Peptide-Nanoparticle System with Improved Efficacy against Multidrug Resistant Bacteria**

Indrani Pal,^[a,b]^ Dipita Bhattacharyya,^[c]^ Rajiv Kumar Kar,^[c]^ D. Zarena^[d]^ Anirban Bhunia,*^[c]^ Hanudatta S. Atreya*^[a]^


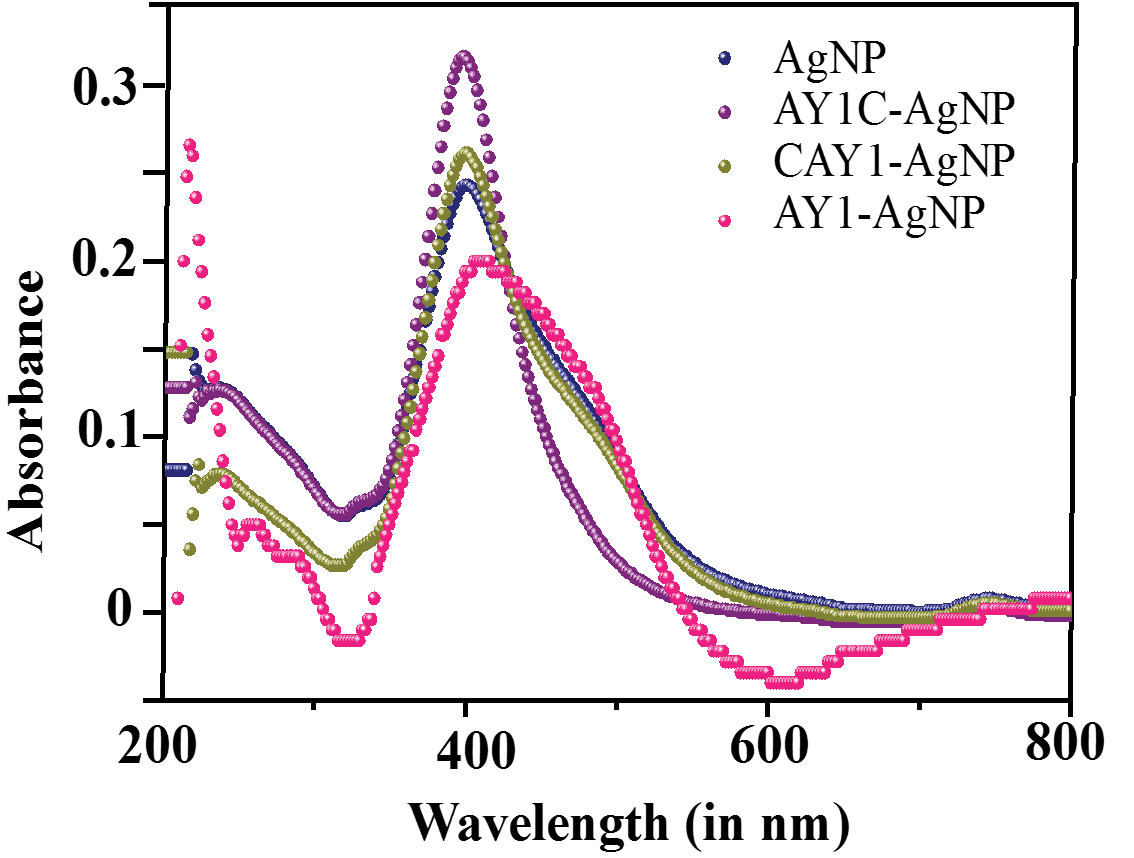


**Figure S1.** Overlay of UV-spectra of AgNP and conjugates


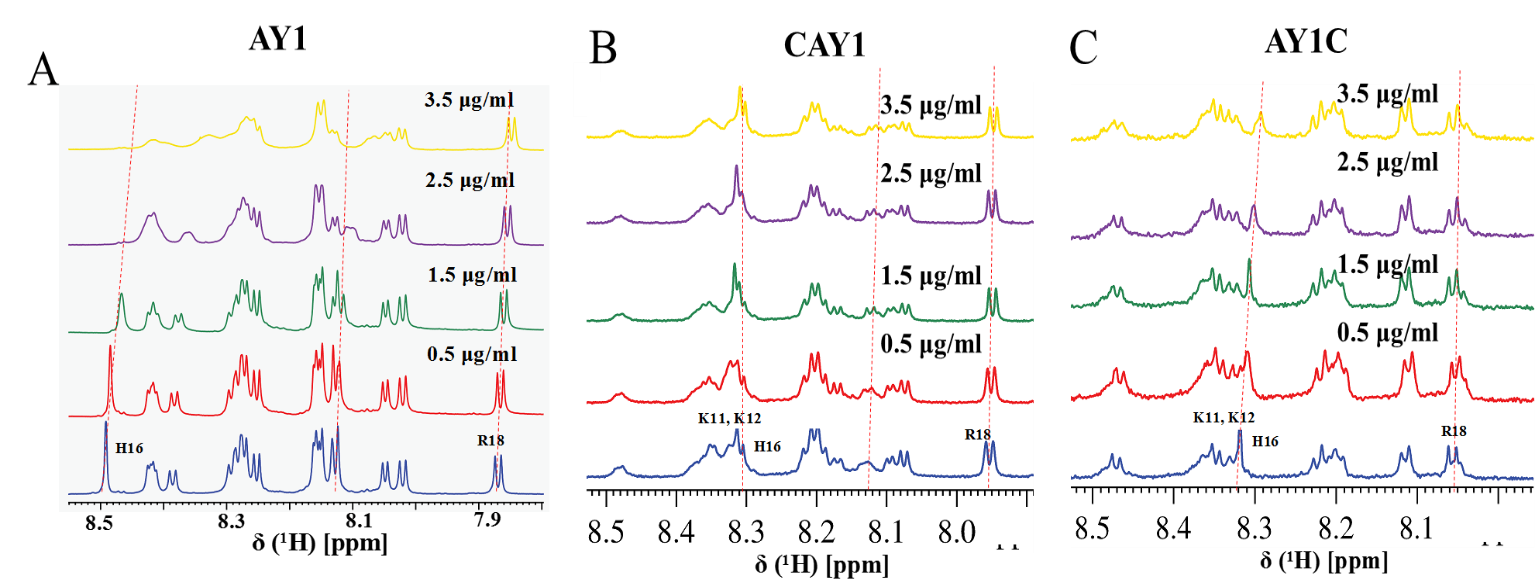


**Figure S2.** A) Stacked plot of ^1^H-NMR spectra acquired for titration of: (a) AY1 with AgNP; b) CAY1 and c) AY1C. The amount of AgNP added at each step of titration is indicated.


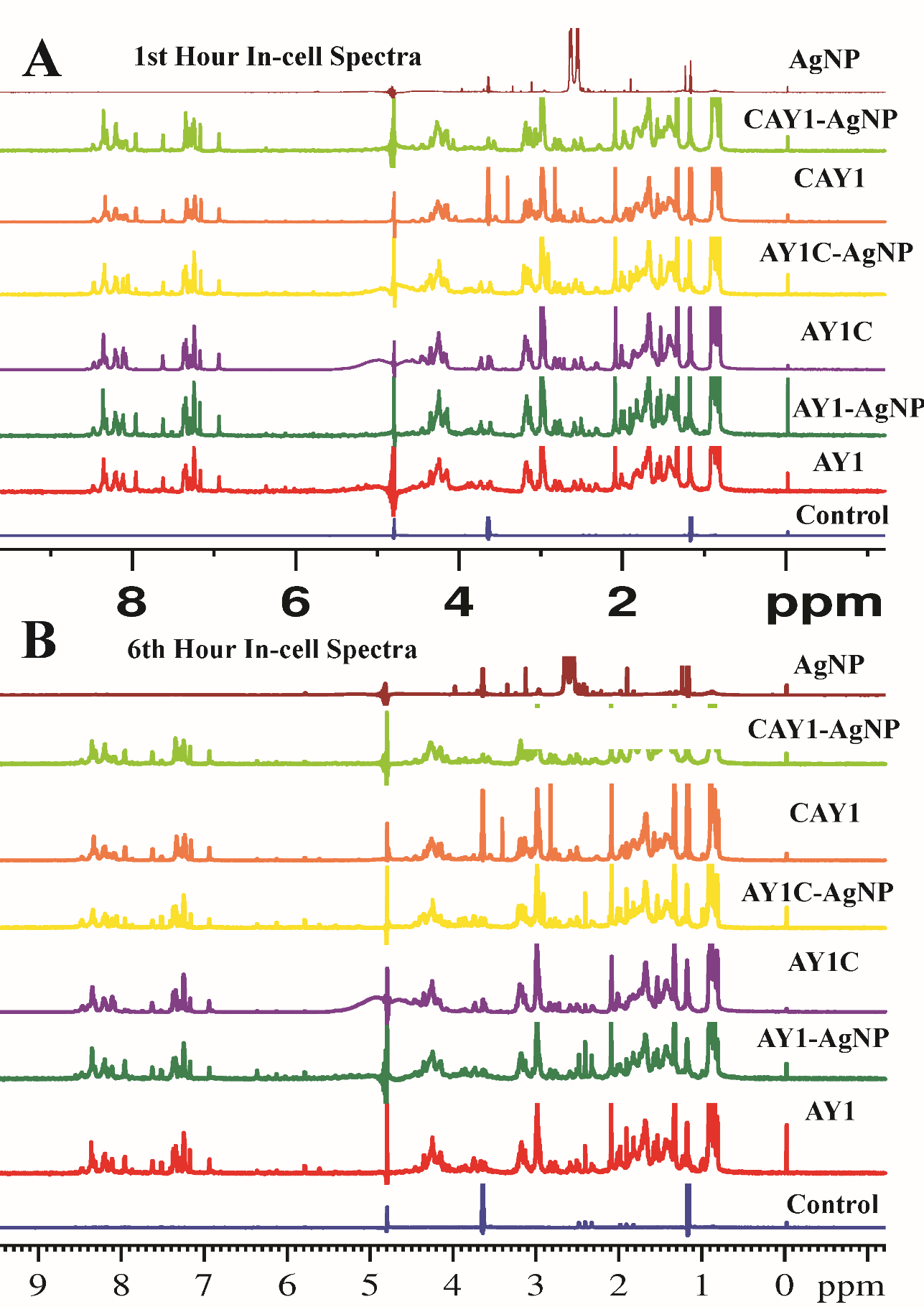


**Figure S3.** In-cell ^1^H-NMR stacked plot of peptides and conjugates at A) 1^st^ hour and B) 6^th^ hour. Notably there is no change in the resonances (signals) of the peptides, indicating that the conjugates remain stable during the entire course of the experiment


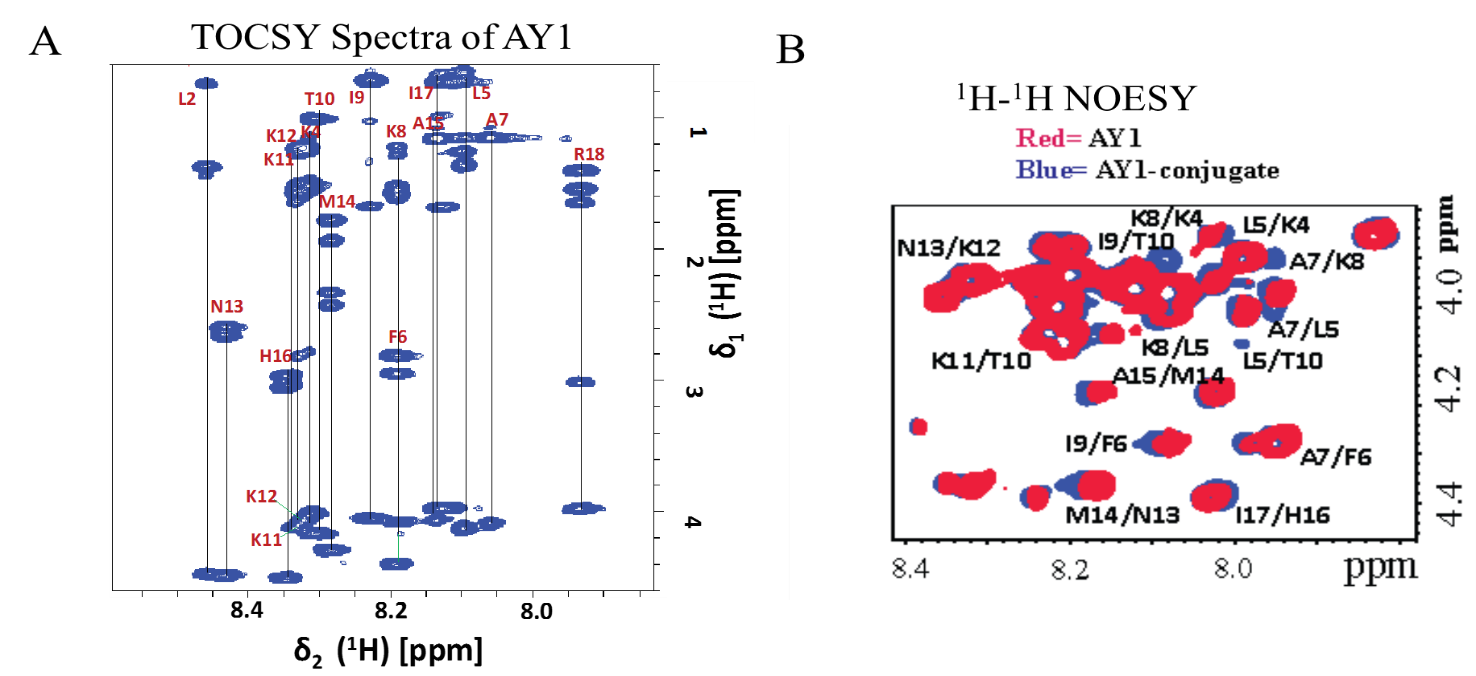


**Figure S4.** The 2D ^1^H-^1^H TOCSY spectra of AY1 in presence of PG/PC (the assignment of resonances are indicated).


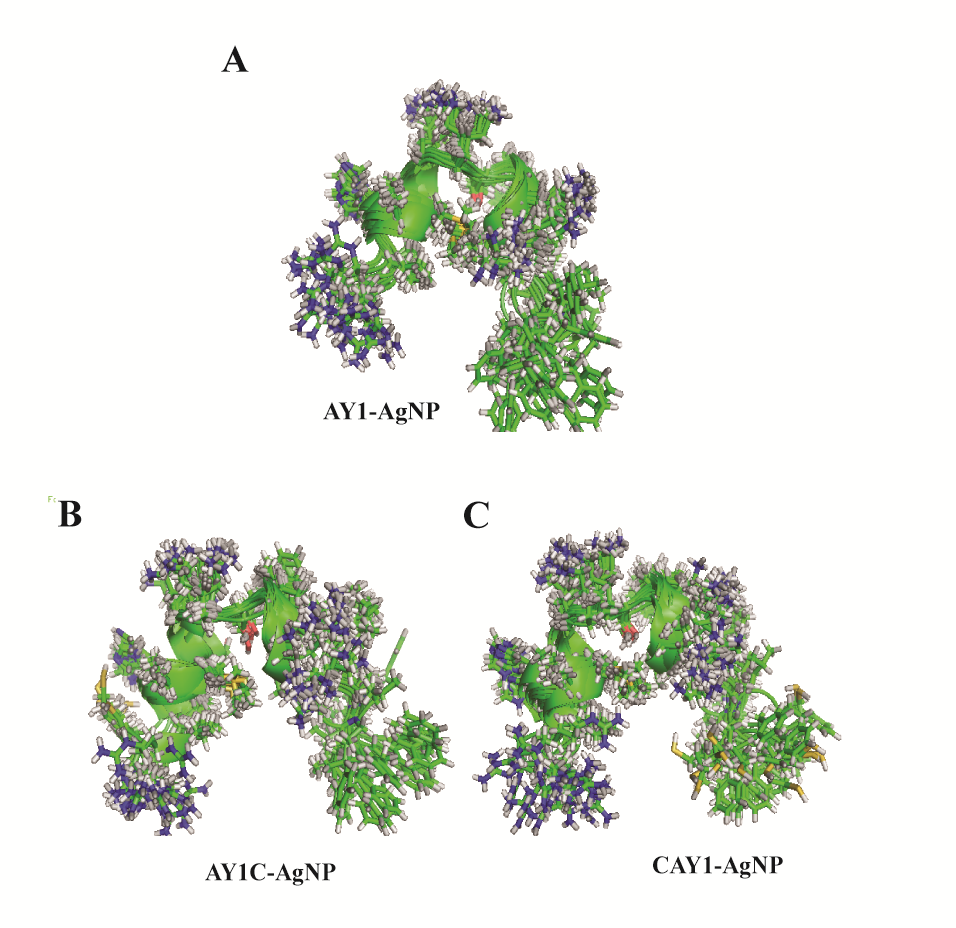


**Figure S5.** 20 structures ensembles of A) AY1, B) AY1C and C) CAY1 in conjugated form.


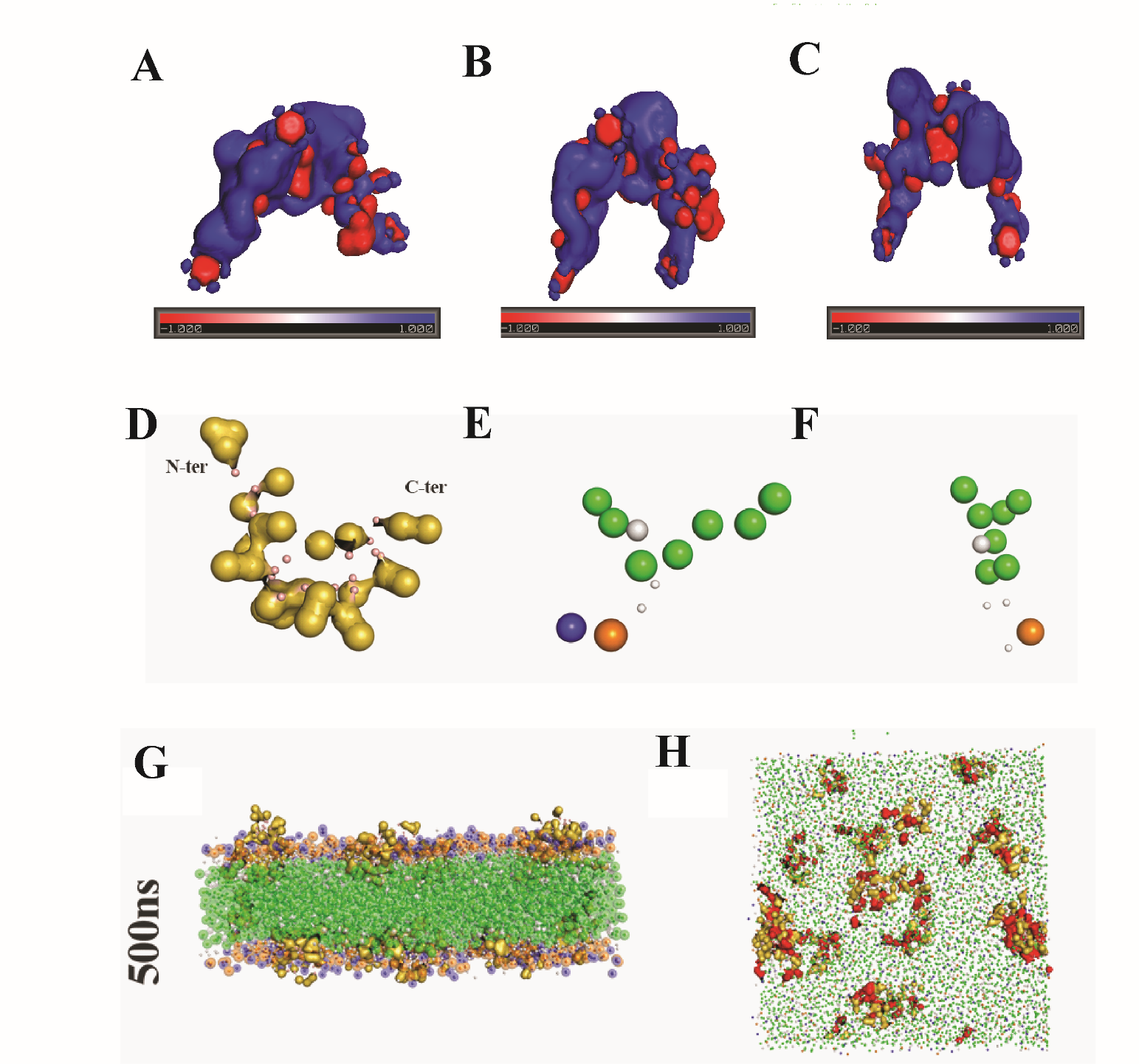


**Figure S6.** Surface charge distribution map in pH=7.4 for A) AY1, B) AY1C, C) CAY1; D) surface representation of CG model for peptide AY1; E) CG model of POPC; F) CG model of POPG; G) A snapshot of MD simulation at 500 ns and H) A cross-section view of MD simulation at 500 ns.

**Table S1.** Structure calculation Statistics

| **Parameters** | AY1-conjugate | AY1C-conjugate | CAY1-conjugate | CAY1 |  |
| --- | --- | --- | --- | --- | --- |
| Residues in most favoured Region of the Ramachandran Plot | 74.60% | 75% | 79.6% | 76.6% |  |
| Residues in additional allowed regions | 25.40% | 25% | 20.4% | 23.4% |  |
| Residues in generously allowed regions | 0% | 0% | 0% | 0% |  |
| Residues in disallowed regions | 0% | 0% | 0% | 0% |  |
| Angular restraints used for structure calculations |  |  |  |  |  |
| Φ | 18 | 19 | 19 | 19 |  |
| Ψ | 18 | 19 | 19 | 19 |  |
| NOE restraints used for structure calculation | 124 | 128 | 122 | 122 |  |
| Distance restraint  violations | 0 | 0 | 0 | 0 |  |
| Average RMSD of backbone (to mean structure) | 0.9 Å ± 0.1 Å | 0.79Å ± 0.1 Å | 0.8Å ± 0.4 Å | 0.78Å ± 0.4 Å |  |
| Average RMSD of heavy atoms | 1.7 Å ± 0.5 Å | 1.29 Å ± 0.5 Å | 1.59 Å ± 0.9 Å | 1.59 Å ± 0.9 Å |  |

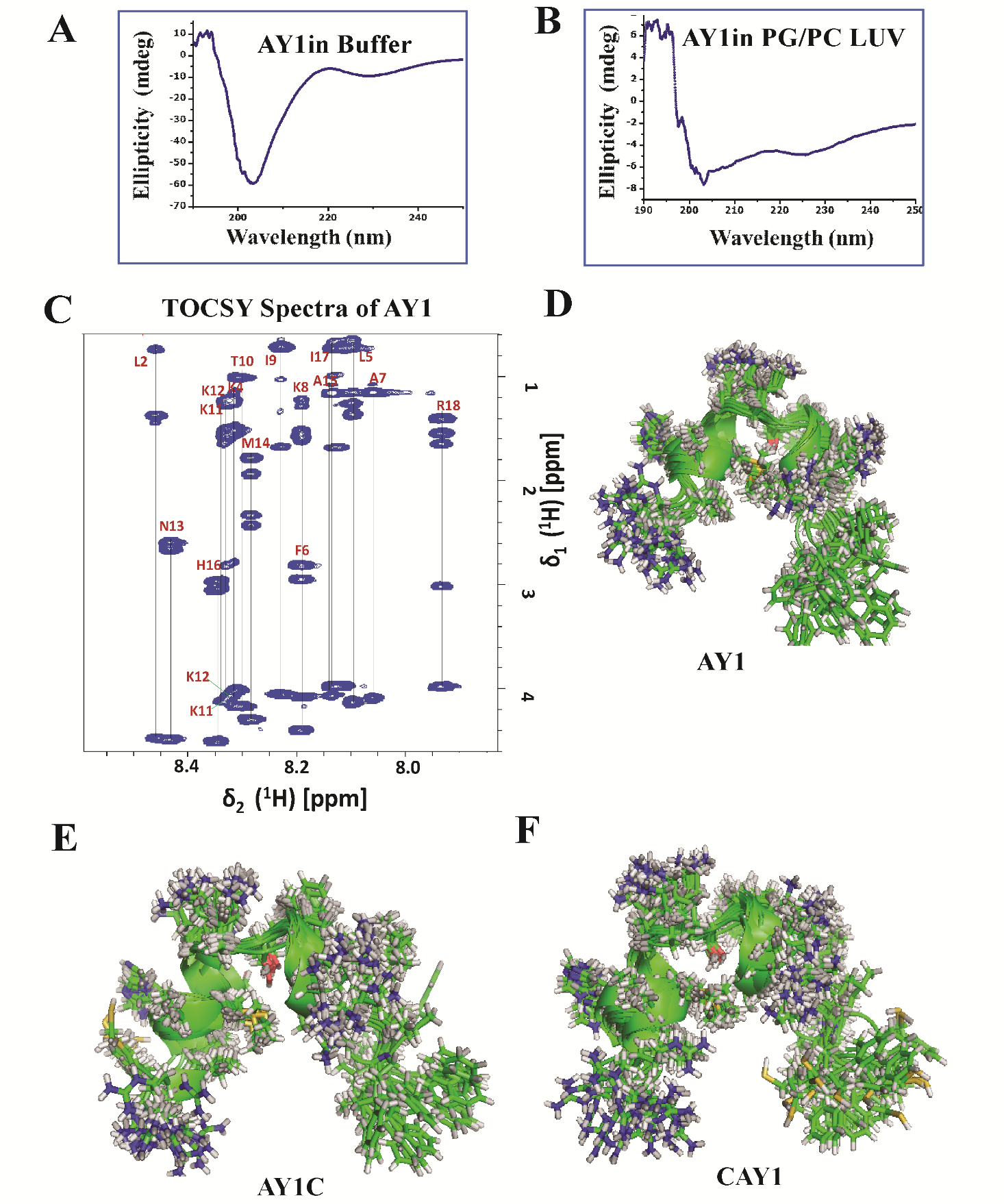
**Figure S7:** **Movie:** CG-MD trajectory, indicative of pore/channel formation by peptide AY1 within the membrane architecture.

**Figure S8:** CD spectra of A) Free AY1 and B) AY1 in presence of PG/PC LUV.

**Table S2:** Data Analysis for Dye-leakage assay

AY1 Std dev CAY1 Std dev AY1C S.d AY1-AgNP S.d. CAY1-AgNP S.d AY1C-AgNP S.d

5 6.07169 0.01 7.54008 0.009 29.56063 0.01 9.87559 0.009 25.37481 0.009 39.40023 0.009

10 12.8 0.1 44.06 0.01 42.58 0.01 13.1243 0.01 35.18887 0.01 47.73762 0.01

20 12.8 0.1 47.81 0.01 47.24 0.01 14.99417 0.01 42.49734 0.01 54.47726 0.01

40 18.28 0.009 48.09 0.009 49.68 0.009 20.70924 0.009 47.80012 0.009 63.44329 0.009

60 18.28 0.009 49.08 0.01 49.68 0.01 23.49052 0.01 51.93311 0.009 66.35903 0.009

80 18.28 0.009 49.08 0.01 49.68 0.01 26.6181 0.01 52.37183 0.01 66.99162 0.01
